# PPAR-*γ*: Therapeutic Potential for Multiple Sclerosis

**DOI:** 10.1155/2008/627463

**Published:** 2008-06-29

**Authors:** Paul D. Drew, Jihong Xu, Michael K. Racke

**Affiliations:** ^1^Department of Neurobiology and Developmental Sciences, University of Arkansas for Medical Sciences, Biomedical Research Building II, Room 563-2, 4301 W. Markham Street, Little Rock, AR 72205, USA; ^2^Department of Neurology, The Ohio State University Medical Center, Columbus, OH 43210, USA

## Abstract

The role of peroxisome proliferator-activated receptors (PPARs) in altering lipid and glucose metabolism is well established. More recent studies indicate that PPARs also play critical roles in controlling immune responses. We and others have previously demonstrated that PPAR-*γ* agonists modulate the development of experimental autoimmune encephalomyelitis (EAE), an animal model of multiple sclerosis (MS). This review will discuss the cellular and molecular mechanisms by which these agonists are believed to modulate disease. The therapeutic potential of PPAR-*γ* agonists in the treatment of multiple sclerosis will also be considered.

## 1. INTRODUCTION

Multiple sclerosis (MS) is the second most common neurologic disorder of young adults, behind neurotrauma. Approximately 350000–400000 individuals have physician diagnosed MS in the United States alone. MS is commonly diagnosed around the third decade of life and many patients suffer the devastating effects of the disease for much of their adult lives. The etiology of MS is not completely understood but is believed to result from a combination of genetic and environmental factors. The disease is characterized by inflammation of the central nervous system (CNS), demyelination, and either relapsing-remitting or progressive clinical presentations. Similarities to experimental autoimmune encephalomyelitis (EAE), an established animal model of MS which is elicited following generation and attack of autoreactive T cells against brain tissues suggests an autoimmune origin for MS. In addition to autoreactive T cells, other peripheral immune cells including B cells, monocytes, and dendritic cells may play a role in the pathogenesis associated with MS. In addition, resident CNS cells including chronically activated glial cells are believed to play a role in disease pathogenesis [1].

Nuclear receptors are a family of transcription factors that regulate gene expression in response to ligand binding. Nuclear receptor superfamily members include peroxisome proliferator-activated receptors (PPARs) as well as androgen, estrogens, progesterone, thyroid, and glucocorticoid receptors. Additional orphan nuclear receptors exist for which ligands have not been identified. The critical role of PPARs in modulating glucose and lipid metabolism has been extensively documented [2]. More recently, a role for PPARs in altering immune responses has been established. A role for PPARs in modulation of immune responses was suggested by the observation that indomethacin, a nonsteroidal anti-inflammatory drug (NSAID) binds PPAR-*γ*. Furthermore, it was documented that PPAR-*γ* is expressed by cells of the monocyte/macrophage lineage. These observations led to seminal studies demonstrating that PPAR-*γ* agonists suppress the activation of monocyte/macrophages [3, 4]. Three PPAR isoforms, PPAR-*α*, -*β*/*δ*, and -*γ*, have been identified. These receptors exhibit distinct tissue expression patterns and ligand specificities [2, 5]. Eicosanoids, polyunsaturated fatty acids, and the cyclopentenone prostaglandin 15d-PGJ_2_ are naturally occurring PPAR-*γ* ligands. Synthetic PPAR-*γ* ligands include thiazolidinediones which are used for the treatment of type II diabetes.

As transcription factors, PPARs primarily function to regulate the expression of specific genes. Similar to other nuclear receptors, PPARs bind DNA and regulate gene expression as dimers. PPARs form heterodimers with retinoid-X-receptors (RXRs), and bind DNA at conserved *peroxisome-proliferator response elements* (PPREs) present in the promoter of PPAR-responsive target genes. Upon ligand binding, the PPAR/RXR heterodimer associates with coactivator complexes, binds PPREs, and activates the transcription of PPAR-responsive genes. In contrast, PPAR/RXR heterodimers not bound by ligand associate with corepressor complexes resulting in suppression of gene transcription [2]. PPAR ligands principally activate transcription of genes encoding proteins important in lipid and glucose metabolism by triggering PPAR/RXR binding to PPREs present in the promoters of these genes. In contrast, PPAR agonists generally suppress the expression of genes encoding proinflammatory molecules through a mechanism not involving PPAR/RXR binding to PPREs. This mechanism, termed receptor-dependent transrepression, is believed to occur through physical interaction between PPAR/RXR and other transcription factors which normally activate transcription of proinflammatory genes. Physical interaction with PPAR/RXR inhibits binding of these transcription factors to response elements present on genes encoding proinflammatory molecules, thus suppressing the activation of these genes. Receptor-dependent transrepression may also result from PPAR/RXR interaction with transcriptional coactivator or corepressor molecules that are in limited supply, or PPAR/RXR interactions with the basal transcription machinery [6, 7]. PPAR-*γ* agonists inhibit transcription factors including NF-*κ*B, AP-1, and STAT-1 from activating gene expression through receptor-dependent transrepression [8]. The mechanisms resulting in receptor-dependent transrepression have remained a mystery. However, recent pioneering work by Glass et al. has begun to elucidate the molecular mechanisms that control receptor-dependent transrepression of NF-*κ*B responsive genes. These studies demonstrate that in the presence of PPAR-*γ* ligands, PPAR-*γ* can conjugate with small ubiquitin-like modifier-1 (SUMO1) resulting in the sumoylation of PPAR-*γ*. Sumoylated PPAR-*γ* binds the corepressor molecule NCoR which maintains the promoters of responsive genes in a repressed state, even in the presence of NF-*κ*B activating stimuli. The mechanisms by which NF-*κ*B responsive genes are believed to remain in a repressed state following sumoylation of PPAR-*γ* and consequent association with NCoR are believed to involve inhibition of the recruitment of ubiquitin conjugating enzymes to the corepressor complex following physical association of sumoylated PPAR-*γ* with NCoR [9–11]. Interestingly, recent studies have demonstrated that in addition to PPAR-*γ*, liver X receptor (LXR) mediated transrepression involves sumoylation of receptor and association of NCoR [12]. This suggests the possibility of a general mechanism of transrepression by PPARs and LXRs.

As stated above, PPAR-*γ* agonists can regulate gene expression in a receptor-dependent manner through receptor binding to PPREs or through receptor-dependent transrepression. In addition, PPAR-*γ* agonists including 15d-PGJ2 can regulate gene expression through receptor-independent mechanisms. For example, 15d-PGJ2 blocks I-*κ*B degradation by inhibiting the activation of I-*κ*B kinase resulting in the retention of NF-*κ*B in the cytoplasm [13, 14]. In addition, 15d-PGJ_2_ has been demonstrated to inhibit NF-*κ*B binding to NF-*κ*B DNA-response elements [15]. Thus, in summary, PPAR-*γ* agonists can regulate gene expression through both receptor-dependent and receptor-independent mechanisms.

## 2. EFFECTS OF PPAR-*γ* ON IMMUNE CELL FUNCTION

### 2.1. CNS resident cells

Microglia are bone marrow-derived cells that migrate to the CNS during embryonic development. Normally, these cells exist in a quiescent state in the CNS. Likewise, astrocytes, resident CNS cells that protect neurons through production of neurotrophic factors as well as uptake of glutamate and other neurotoxic molecules, commonly are quiescent in the CNS. However, these glial cells may become activated in response to insults including stress, trauma, and pathogens, and under these conditions may initiate protective immune responses. Upon activation, microglia and astrocytes produce proinflammatory molecules including nitric oxide (NO), cytokines, and chemokines. These proinflammatory molecules play critical roles in removing pathogens and debris from the infected or injured CNS. In contrast, chronically activated glia are believed to contribute to CNS damage characteristic of neuroinflammatory and neurodegenerative disorders including MS.

Van Eldik et al. were the first to evaluate the effects of PPAR-*γ* agonists on immune function in glial cells. They demonstrated that the PPAR-*γ* agonist 15d-PGJ_2_ inhibited LPS induction of NO and iNOS expression in the murine BV-2 microglial cell line. However, troglitazone which is a PPAR-*γ* agonist and thiazolidinedione did not suppress LPS induction of these molecules. These results were interpreted to indicate that 15d-PGJ_2_ functioned through a receptor-independent mechanism. Using the same BV-2 microglial cell system, the Van Eldik laboratory also demonstrated that 15d-PGJ_2_ suppressed LPS induction of TNF-*α*, IL-1*β*, and COX-2 [16]. However, 15d-PGJ_2_ increased intracellular glutathione levels as well as expression of heme oxygenase-1, an enzyme known to stimulate antioxidant production [17]. Minghetti et al. were the first to investigate the effects of PPAR-*γ* agonists on immune function in primary microglia. These studies indicated that 15d-PGJ_2_ as well as the thiazolidinedione ciglitazone suppressed LPS induction of iNOS and TNF-*α* expression by primary rat microglia. These PPAR-*γ* agonists also suppressed IFN-*γ* induction of major histocompatibility class II in these cells. Because 15d-PGJ_2_ and ciglitazone effects on microglial immune cell function were similar in these studies, it was interpreted that 15d-PGJ_2_ functioned through a receptor-dependent mechanism [18].

More recently, we compared the effects of a series of thiazolidinediones or 15d-PGJ_2_ on the production of proinflammatory molecules by primary microglia and astrocytes. These studies demonstrated that both thiazolidinediones and 15d-PGJ_2_ inhibited the production of NO, the proinflammatory cytokines TNF-*α*, IL-1*β*, and IL-6, and the chemokine MCP-1 by these glial cells [19]. However, even though 15d-PGJ_2_ binds PPAR-*γ* with less affinity than each of the thiazolidinediones, this cyclopentenone prostaglandin more strongly inhibited production of these proinflammatory molecules by the glial cells, suggesting that 15d-PGJ_2_ acts at least in part through receptor-independent mechanisms. A receptor-dependent effect of 15d-PGJ_2_ in regulating glial cell immune function is supported by studies demonstrating that microglial cell activation is suppressed in a cooperative manner by this PPAR-*γ* ligand in combination with 9-cis retinoic acid, the ligand for the retinoic acid receptor RXR [20]. This supports the hypothesis that glial cell activation is maximally suppressed in the presence of ligands following formation of PPAR-*γ*/RXR heterodimers. Our observation that monocyte/microglia and astrocytes express increased levels of PPAR-*γ* during active EAE suggests that this receptor may modulate disease, perhaps through effects on glial cell activation [21]. Luna-Medina et al. [22] demonstrated that thiazolidinediones inhibited LPS induction of proinflammatory molecules by microglia and astrocytes. In addition, thiazolidinedione treatment of glial cultures suppressed the production of neurotoxic molecules. The effects of thiazolidinediones on glia in these studies were abrogated by PPAR-*γ* antagonists suggesting a receptor-dependent mechanism [22]. Minghetti et al. demonstrated that two flurbiprofen derivatives demonstrated to release NO suppressed glial cell activation through activation of PPAR-*γ* [23, 24]. Interestingly, one of these compounds, NXC 2216, initially activated the receptor, but later stimulated nitration and inactivation of PPAR-*γ* [24]. This suggested that these flurbiprofen derivatives could differentially activate or suppress glial immune cell function depending on length of treatment. Interestingly, PGA_2_ potently suppressed microglia and astrocyte production of proinflammatory molecules [25]. Structurally, PGA_2_ is a cyclopentenone prostaglandin like 15d-PGJ_2_. However, PGA2 is not believed to bind PPAR-*γ*, suggesting that the cyclopentenone ring structure itself may modulate glial cell activation.

Astrocytes, like microglia, react to pathogens through a series of pattern recognition receptors which stimulate toll-like receptor signaling [26, 27]. Kielian et al. demonstrated that both 15d-PGJ_2_ and the thiazolidinedione ciglitazone suppressed Staphylococcus aureus induction of NO and IL-1*β* by primary astrocytes. Suppression of these proinflammatory molecules by 15d-PGJ_2_ and ciglitazone occurred in both wild-type and PPAR-*γ* deficient astrocytes, suggesting that these compounds mediated their effects in a receptor-independent manner [28].

PPAR-*γ* agonists have been demonstrated to modulate a variety of signaling pathways. Singh et al. showed that 15d-PGJ_2_ inhibited LPS induction of NO and proinflammatory molecules in primary astrocytes. Transfection of wild-type PPAR-*γ*, dominant-negative PPAR-*γ*, or treatment of cells with a PPAR-*γ* antagonist did not alter 15d-PGJ_2_ effects on astrocytes in these studies suggesting that the agonist functioned through a receptor-independent mechanism. The PPAR-*γ* agonist decreased NF-*κ*B activity in these studies, presumably by inhibiting I-*κ*B kinase. Singh et al. also demonstrated that 15d-PGJ_2_ inhibited the phosphatidylinositol 3-kinase-Akt signaling pathway, suggesting an additional mechanism by which the agonist inhibited production of proinflammatory molecules in astrocytes [29]. Additional studies indicated that both the thiazolidinedione ciglitazone and 15d-PGJ_2_ stimulated MAP kinase pathways in astrocytes through receptor-independent mechanisms involving production of reactive oxygen species [30]. The PPAR-*γ* agonists rosiglitazone and 15d-PGJ_2_ also were demonstrated to modulate the JAK/STAT pathway by inhibiting the phosphorylation of specific JAK and STAT molecules following induced expression of suppressor of cytokine signaling (SOCS) 1 and 3 proteins in astrocytes and microglia [31].

Collectively, the studies discussed above suggest that PPAR-*γ* agonists may regulate immune function in glia through receptor-dependent or alternatively through receptor-independent mechanisms. Factors that may determine if receptor-dependent or receptor-independent mechanisms are employed may include the specific PPAR-*γ* agonist studied, the concentration of the agonist used, and the cell type studied. For example, responses are likely to differ between primary and transformed cells and may vary depending on the developmental state of the tissue from which the glial cells are derived.

The function and phenotype of T cells can be dramatically altered by glia. For example, the IL-12 family of cytokines which includes IL-12, IL-23, and IL-27 is believed to alter T cell phenotype and modulate the development of EAE and MS [32]. Specifically, the IL-12 family of cytokines modulates the differentiation of Th_1_ cells which are believed to contribute to the development of EAE. In addition, IL-23 contributes to the production of Th_17_ cells which have recently been demonstrated to play a critical role in autoimmunity. IL-12 family members are heterodimeric. IL-12 consists of a dimer of p40 and p35 subunits. IL-23 consists of the same p40 subunit in association with p19. IL-27 exists as a dimer of p28 in association with EBV-induced molecule 3 (EBI3). Previously, we demonstrated that the PPAR-*γ* agonist 15d-PGJ_2_ potently inhibited LPS induction of IL-12 p40 secretion by N9 mouse microglial cells and primary rat microglia [33]. More recently, we demonstrated that 15d-PGJ_2_ and the thiazolidinedione rosiglitazone inhibited LPS induction of IL-12 p40, IL-12 p70, IL-23, and IL-27 p28 in primary microglia. In addition, 15d-PGJ_2_ inhibited IL-12 p40, IL-23, and IL-27 p28, while rosiglitazone inhibited IL-23, and IL-27 p28, but not IL-12 p40 in primary astrocytes. LPS did not stimulate the production of IL-12 p70 in astrocytes [34]. These studies suggest that PPAR-*γ* agonists may modulate the development of EAE in part by modulating IL-12 family cytokine production by glia, which may alter T cell phenotype. Costimulatory molecules may be expressed by antigen presenting cells (APCs) including CNS microglia. Interaction of costimulatory molecules including CD40, CD80, and CD86 on APCs with their cognate receptors present of CD4^+^ T cells is important in the activation and differentiation of these T cells, which likewise modulate the development of EAE and possibly MS. Our previous studies indicated that 15d-PGJ_2_ inhibited microglial expression of CD40, but had no effect on the expression of CD80 and CD86 costimulatory molecules. Therefore, through modulation of costimulatory molecule expression by microglia, PPAR-*γ* agonists may alter the pathogenesis of EAE [21, 35].

PPAR-*γ* agonists can alter the viability of neurons and oligodendrocytes, which are CNS cells compromised in MS. These agonists may alter the viability of these cells directly or indirectly by suppressing the production of cytotoxic molecules by activated microglia and astrocytes. Combs et al. [36] demonstrated that treatment of glial cultures with a variety of PPAR-*γ* agonists suppressed *β*-amyloid mediated toxicity of cortical neurons. Similarly, PPAR-*γ* agonists including thiazolidinediones and 15d-PGJ_2_ inhibited LPS induction of neuronal cell death in studies utilizing a rat cortical neuron-glial coculture paradigm [37]. Furthermore, more recent studies indicated that thiazolidinediones treatment of cortical neuron-mixed glia cocultures resulted in protection of neurons. Neuron protection was abrogated in these studies by a PPAR-*γ* antagonist suggesting that neuron protection occurred by a receptor-dependent mechanism [22]. In addition to protecting neurons through suppression of glial activation, PPAR-*γ* agonists can also directly protect neurons. For example, PPAR-*γ* agonists have been demonstrated to protect neurons from a variety of neurotoxic agents including NMDA [38] and apolipoprotein E4 [39]. Interestingly, neurons express PPAR-*γ* and several studies suggest that neuron cell viability may be mediated through PPAR-*γ* activation [40, 41]. However, the mechanisms by which PPAR-*γ* regulates neuron cell viability have not been fully elucidated. Recent studies suggest that one mechanism by which PPAR-*γ* agonists may modulate neuron cell viability is through modulation of the antiapoptotic factor Bcl-2 [42]. Interestingly, PPAR-*γ* also regulates neural stem cell proliferation and differentiation [43]. Less is known concerning the role of PPAR-*γ* in modulating oligodendrocyte cell viability and differentiation. However, studies suggest that PPAR-*γ* protects oligodendrocyte progenitors [44] and modulates oligodendrocyte differentiation [45].

### 2.2. Peripheral immune cells

As mentioned previously, autoreactive T cells are believed to contribute to MS pathogenesis. Clark et al. initially demonstrated that T cells express PPAR-*γ* and that 15d-PGJ2 and ciglitazone inhibited T cell secretion of IL-2 and altered T cell proliferation [46]. PPAR-*γ* agonists have been demonstrated to induce apoptosis of T cells [47]. However, others studies indicate that PPAR-*γ* agonists promote the survival of T cells [48]. The exact reason for the discrepancy between these studies is not clear, but may involve differences in the concentration of PPAR-*γ* agonists used in the studies. Regulatory T cells play a critical role in suppressing the development of autoimmune diseases. Interestingly, recent studies indicate that PPAR-*γ* agonists enhance the generation and function of regulatory T cells [49, 50]. It is now clear that B cells have a significant role in modulating EAE and MS. Phipps et al. demonstrated that B cells express PPAR-*γ* and that PPAR-*γ* agonists stimulate the apoptosis of these cells [51, 52]. Collectively, these studies suggest that PPAR-*γ* may modulate MS in part by altering the viability and function of lymphocytes.

As stated previously, macrophages express PPAR-*γ* and PPAR-*γ* agonists regulate the function of these cells [53]. Like macrophages, dendritic cells are also of monocytic origin and function as professional antigen presenting cells. Dendritic cells also play a significant role in MS [54, 55]. Interestingly, PPAR-*γ* agonists have been shown to alter the viability and function of dendritic cells [56]. For example, cyclopentenone prostaglandins induced the apoptosis of dendritic cells, although apoptosis occurred through a receptor-independent mechanism [57]. PPAR-*γ* agonists were also shown to inhibit the migration of dendritic cells [58]. Furthermore, PPAR-*γ* agonists inhibited toll-like receptor mediated activation of dendritic cells by suppressing MAP kinase and NF-*κ*B signaling pathways [59]. PPAR-*γ* effects on dendritic cell function have also been demonstrated to contribute to the development of CD4^+^ T cell anergy [60]. Collectively, these studies indicate that PPAR-*γ* agonists may modulate MS in part through effects on monocytic cells.

Activated peripheral immune cells including antigen specific T cells and macrophages are capable of entering the CNS and contributing to MS pathology. Extravasation of peripheral immune cells is mediated by a variety of factors including chemokines and adhesion molecule expression on the cerebral vascular endothelium. Chemokines are synthesized under inflammatory conditions and generate a concentration gradient to which cells with the appropriate chemokine receptors migrate. The expression of specific chemokines is increased in EAE and MS [61, 62]. PPAR-*γ* agonists decrease the expression of MCP-1 which is a chemoattractant for monocytes and microglia [63, 64] as well as the T cell chemoattractants IP-10 (CXCL3), Mig (CXCL3), and I-TAC (CXCL3) [65]. Adhesion molecules present on the cerebral vascular endothelium facilitate extravasation of peripheral immune cells into the CNS. PPAR-*γ* agonists modulate the expression of various specific adhesion molecules suggesting an additional mechanism controlling immune cell extravasation into the CNS [66–68]. Future studies will be important in determining more detailed mechanisms by which PPAR-*γ* agonists modulate immune cell movement into the CNS.

## 3. EFFECTS OF PPAR-*γ* AGONISTS ON EAE AND MULTIPLE SCLEROSIS

### 3.1. EAE

EAE is a well-established animal model of MS. The disease is induced following immunization of CNS antigens, is mediated by myelin-specific T cells, and is characterized by CNS inflammation, demyelination, and remittent paralysis [1]. The blood-brain-barrier is believed compromised in EAE. However, the relative bioavailability of PPAR-*γ* agonists into the CNS varies, and it is important to consider this variable when interpreting studies designed to evaluate the effects on these agonists on EAE [69, 70]. The effects of PPAR-*γ* agonists in modulating EAE were first investigated by Niino et al. who demonstrated that the thiazolidinedione troglitazone inhibited the development of EAE elicited by MOG_35–55_ immunization of C57BL/6 mice. Troglitazone did not alter T cell proliferation or T cell production of IFN-*γ* in vitro in these studies [71]. We demonstrated that 15d-PGJ_2_ inhibited the proliferation of splenic MBP_Ac1-11_ transgenic T cells and inhibited IL-4 and IFN-*γ* production by these cells in vitro [21]. In vitro treatment of these transgenic T cells with 15d-PGJ_2_ decreased the encephalitogenicity of these cells following adoptive transfer into naïve mice. This PPAR-*γ* agonist also inhibited the development of EAE when administered prior to or following onset of disease in an active model of disease involving immunization of B10.PL mice with MBP_Ac1-11_ [21]. These studies suggest that PPAR-*γ* agonists may be effective in the treatment of established MS. Feinstein et al. demonstrated that monophasic EAE was inhibited by thiazolidinediones including pioglitazone. Although pioglitazone had no effect on the initial phase of relapsing-remitting EAE, disease severity was reduced upon subsequent relapses. In addition, these studies demonstrated that pioglitazone protected against axonal demyelination [72]. Bright et al. demonstrated that the PPAR-*γ* agonists 15d-PGJ_2_ and ciglitazone decreased IL-12 expression and differentiation of Th_1_ cells which was associated with decreased severity of active and passive EAE [73]. This team of investigators later showed that heterozygous PPAR-*γ* deficient mice demonstrated more severe EAE than wild-type mice [74]. They also demonstrated that more severe EAE developed following treatment with PPAR-*γ* antagonists [75]. We showed that a combination of the PPAR-*γ* agonist 15d-PGJ_2_ and the RXR agonist 9-cis retinoic acid cooperatively inhibited the development of EAE [20]. Collectively, these studies support a role for PPAR-*γ* in modulating EAE. Homozygous PPAR-*γ* mutations are lethal thus complicating additional studies designed to evaluate the role of this receptor in modulating disease. Development of conditional PPAR-*γ* knockout mice as well as highly specific PPAR-*γ* antagonists will help define the role of PPAR-*γ* in modulation of EAE.

### 3.2. MS

Heneka et al. investigated the effects of thiazolidinediones pioglitazone and ciglitazone and the nonthiazolidinedione PPAR-*γ* agonist GW347845 on the function of peripheral blood mononuclear cells (PBMCs) from MS patients and healthy donors. These studies demonstrated that all of these PPAR-*γ* agonists decreased phytohemagglutinin (PHA) induced T cell proliferation and production of the cytokines TNF-*α* and IFN-*γ* by PBMCs. Interestingly, proliferation and cytokine secretion were further suppressed following pretreatment of cells with PPAR-*γ* agonists. These studies also demonstrated that the PPAR-*γ* agonists decreased bcl-2 expression and induced apoptosis of activated T cells [76]. Additional studies by the same group indicated that PPAR-*γ* expression was reduced in PBMCs from MS patients relative to healthy donors, which correlated with decreased anti-inflammatory effects of pioglitazone on patient-derived PBMCs [77]. PHA stimulation of PBMCs from healthy donors resulted in decreased PPAR-*γ* expression, which was overcame by pretreatment of these cells with PPAR-*γ* agonist. Long-term treatment of diabetes patients with pioglitazone also overcame the decrease in PPAR-*γ* expression in PHA treated PBMCs from these patients. These studies indicate that pioglitazone treatment in humans can protect against loss of PPAR-*γ* expression resulting from inflammation. These studies also indicated that preincubation of PBMCs with pioglitazone resulted in increased PPAR-*γ* and decreased NF-*κ*B DNA-binding activity [77]. Collectively, these studies suggest that sustained activation of PPAR-*γ* may prevent inflammation induced reduction of the expression of this receptor. These studies may have important implications concerning the use of PPAR-*γ* agonists in the treatment of MS.

A small clinical study supports the idea that PPAR-*γ* agonists may be effective in the treatment of MS [78]. Larger scale clinical trials are currently underway to further assess the therapeutic potential of PPAR-*γ* agonists for the treatment of MS.

## 4. CONCLUSIONS

PPAR-*γ* agonists have been demonstrated to limit pathology in animal models of human neuroinflammatory and neurodegenerative disorders. These studies suggest that these agonists may be effective in the treatment of human diseases including MS. Type II diabetes is commonly treated with PPAR-*γ* agonists termed thiazolidinediones which include pioglitazone and rosiglitazone. These medications have an excellent safety profile which should facilitate future clinical trials designed to evaluate the efficacy of these PPAR-*γ* agonists in the treatment of human disorders of the CNS. However, basic research designed to better understand the cellular and molecular mechanisms by which PPAR-*γ* agonists regulate CNS inflammation will be critical in developing more effective treatment strategies for neuroinflammatory disorders including MS.

## Figures and Tables

**Figure 1:**
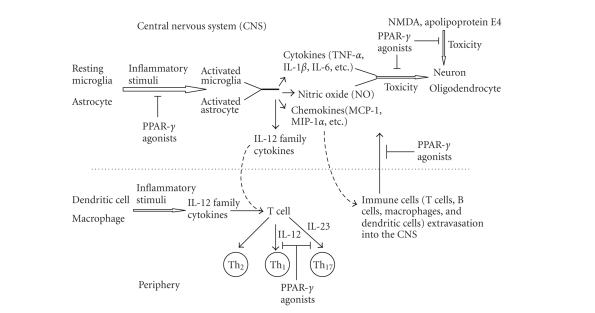
Effects of PPAR-*γ* on immune cell function. Glial cells including microglia and astrocytes may become activated in response to inflammatory stimuli including stress, trauma, and pathogens. Upon activation, microglia, and astrocytes produce a wide range of cytokines as well as NO. These molecules may be toxic to CNS cells, including myelin-producing oligodendrocytes and neurons, which are compromised in the course of MS. PPAR-*γ* agonists block the activation of glial cells resulting in repression of production of cytokines and NO, and protect oligodendrocytes and neurons from the toxic effects of these molecules. PPAR-*γ* agonists can also directly protect neurons from a variety of neurotoxic agents including NMDA and apolipoprotein E4. Chemokines secreted by activated glia cells establish a concentration gradient to which target cell populations migrate, and play important roles in recruiting cells into inflammatory sites in the CNS. PPAR-*γ* agonists may regulate the extravasation of peripheral immune cells into the CNS by suppressing chemokine expression. In addition, activated microglia serve as the major antigen-presenting cells (APCs) in the CNS, and dendritic cells and macrophages serve as APCs in the periphery. These APCs are capable of secreting IL-12 family cytokines upon activation. IL-12 and IL-23 play a critical role in the development of Th_1_ and Th_17_ cells. PPAR-*γ* agonists may inhibit Th_1_ and Th_17_ cell production by repressing IL-12 family cytokine secretion by these APCs, which is believed to protect against EAE/MS.

## References

[1] Martin R, McFarland HF, McFarlin DE (1992). Immunological aspects of demyelinating diseases. *Annual Review of Immunology*.

[2] Desvergne B, Wahli W (1999). Peroxisome proliferator-activated receptors: nuclear control of metabolism. *Endocrine Reviews*.

[3] Jiang C, Ting AT, Seed B (1998). PPAR-*γ* agonists inhibit production of monocyte inflammatory cytokines. *Nature*.

[4] Ricote M, Li AC, Willson TM, Kelly CJ, Glass CK (1998). The peroxisome proliferator-activated receptor-*γ* is a negative regulator of macrophage activation. *Nature*.

[5] Straus DS, Glass CK (2001). Cyclopentenone prostaglandins: new insights on biological activities and cellular targets. *Medicinal Research Reviews*.

[6] Kamei Y, Xu L, Heinzel T (1996). A CBP integrator complex mediates transcriptional activation and AP-1 inhibition by nuclear receptors. *Cell*.

[7] Kerppola TK, Luk D, Curran T (1993). Fos is a preferential target of glucocorticoid receptor inhibition of AP-1 activity in vitro. *Molecular and Cellular Biology*.

[8] Li M, Pascual G, Glass CK (2000). Peroxisome proliferator-activated receptor *γ*-dependent repression of the inducible nitric oxide synthase gene. *Molecular and Cellular Biology*.

[9] Pascual G, Fong AL, Ogawa S (2005). A SUMOylation-dependent pathway mediates transrepression of inflammatory response genes by PPAR-*γ*. *Nature*.

[10] Ricote M, Glass CK (2007). PPARs and molecular mechanisms of transrepression. *Biochimica et Biophysica Acta*.

[11] Straus DS, Glass CK (2007). Anti-inflammatory actions of PPAR ligands: new insights on cellular and molecular mechanisms. *Trends in Immunology*.

[12] Ghisletti S, Huang W, Ogawa S (2007). Parallel SUMOylation-dependent pathways mediate gene- and signal-specific transrepression by LXRs and PPAR*γ*. *Molecular Cell*.

[13] Castrillo A, Díaz-Guerra MJM, Hortelano S, Martín-Sanz P, Boscá L (2000). Inhibition of I*κ*B kinase and I*κ*B phosphorylation by 15-deoxy-Δ^12,14^-prostaglandin J_2_ in activated murine macrophages. *Molecular and Cellular Biology*.

[14] Rossi A, Kapahi P, Natoli G (2000). Anti-inflammatory cyclopentenone prostaglandins are direct inhibitors of I*κ*B kinase. *Nature*.

[15] Straus DS, Pascual G, Li M (2000). 15-deoxy-Δ^12,14^-prostaglandin J_2_ inhibits multiple steps in the NF-*κ*B signaling pathway. *Proceedings of the National Academy of Sciences of the United States of America*.

[16] Petrova TV, Akama KT, Van Eldik LJ (1999). Cyclopentenone prostaglandins suppress activation of microglia: down-regulation of inducible nitric-oxide synthase by 15-deoxy-Δ^12,14^-prostaglandin J_2_. *Proceedings of the National Academy of Sciences of the United States of America*.

[17] Koppal T, Petrova TV, Van Eldik LJ (2000). Cyclopentenone prostaglandin 15-deoxy-Δ^12,14^-prostaglandin J_2_ acts as a general inhibitor of inflammatory responses in activated BV-2 microglial cells. *Brain Research*.

[18] Bernardo A, Levi G, Minghetti L (2000). Role of the peroxisome proliferator-activated receptor-*γ* (PPAR-*γ*) and its natural ligand 15-deoxy-Δ^12,14^-prostaglandin J_2_ in the regulation of microglial functions. *European Journal of Neuroscience*.

[19] Storer PD, Xu J, Chavis J, Drew PD (2005). Peroxisome proliferator-activated receptor-gamma agonists inhibit the activation of microglia and astrocytes: implications for multiple sclerosis. *Journal of Neuroimmunology*.

[20] Diab A, Hussain RZ, Lovett-Racke AE, Chavis JA, Drew PD, Racke MK (2004). Ligands for the peroxisome proliferator-activated receptor-*γ* and the retinoid X receptor exert additive anti-inflammatory effects on experimental autoimmune encephalomyelitis. *Journal of Neuroimmunology*.

[21] Diab A, Deng C, Smith JD (2002). Peroxisome proliferator-activated receptor-*γ* agonist 15-deoxy-Δ^12,14^-prostaglandin J_2_ ameliorates experimental autoimmune encephalomyelitis. *Journal of Immunology*.

[22] Luna-Medina R, Cortes-Canteli M, Alonso M, Santos A, Martínez A, Perez-Castillo A (2005). Regulation of inflammatory response in neural cells in vitro by thiadiazolidinones derivatives through peroxisome proliferator-activated receptor *γ* activation. *Journal of Biological Chemistry*.

[23] Bernardo A, Ajmone-Cat MA, Gasparini L, Ongini E, Minghetti L (2005). Nuclear receptor peroxisome proliferator-activated receptor-*γ* is activated in rat microglial cells by the anti-inflammatory drug HCT1026, a derivative of flurbiprofen. *Journal of Neurochemistry*.

[24] Bernardo A, Gasparini L, Ongini E, Minghetti L (2006). Dynamic regulation of microglial functions by the non-steroidal anti-inflammatory drug NCX 2216: implications for chronic treatments of neurodegenerative diseases. *Neurobiology of Disease*.

[25] Storer PD, Xu J, Chavis JA, Drew PD (2005). Cyclopentenone prostaglandins PGA_2_ and 15-deoxy-*δ*
^12,14^PGJ_2_ suppress activation of marine microglia and astrocytes: implications for multiple sclerosis. *Journal of Neuroscience Research*.

[26] Kielian T (2006). Toll-like receptors in central nervous system glial inflammation and homeostasis. *Journal of Neuroscience Research*.

[27] Farina C, Aloisi F, Meinl E (2007). Astrocytes are active players in cerebral innate immunity. *Trends in Immunology*.

[28] Phulwani NK, Feinstein DL, Gavrilyuk V, Akar C, Kielian T (2006). 15-deoxy-Δ^12,14^-prostaglandin J2 (15d-PGJ2) and ciglitazone modulate *Staphylococcus aureus*-dependent astrocyte activation primarily through a PPAR-*γ*-independent pathway. *Journal of Neurochemistry*.

[29] Giri S, Rattan R, Singh AK, Singh I (2004). The 15-deoxy-*δ*
^12,14^-prostaglandin J_2_ inhibits the inflammatory response in primary rat astrocytes via down-regulating multiple steps in phosphatidylinositol 3-kinase-akt-NF-*κ*B-p300 pathway independent of peroxisome proliferator-activated receptor *γ*. *Journal of Immunology*.

[30] Lennon AM, Ramaugé M, Dessouroux A, Pierre M (2002). MAP kinase cascades are activated in astrocytes and preadipocytes by 15-deoxy-Δ^12,14^-prostaglandin J_2_ and the thiazolidinedione ciglitazone through peroxisome proliferator activator receptor *γ*-independent mechanisms involving reactive oxygenated species. *Journal of Biological Chemistry*.

[31] Park EJ, Park SY, Joe E-H, Jou I (2003). 15d-PGJ_2_ and rosiglitazone suppress Janus kinase-STAT inflammatory signaling through induction of suppressor of cytokine signaling 1 (SOCS1) and SOCS3 in glia. *Journal of Biological Chemistry*.

[32] Gran B, Zhang G-X, Rostami A (2004). Role of the IL-12/IL-23 system in the regulation of T-cell responses in central nervous system inflammatory demyelination. *Critical Reviews in Immunology*.

[33] Drew PD, Chavis JA (2001). The cyclopentone prostaglandin 15-deoxy-Δ^12,14^ prostaglandin J_2_ represses nitric oxide, TNF-*α*, and IL-12 production by microglial cells. *Journal of Neuroimmunology*.

[34] Xu J, Drew PD (2007). Peroxisome proliferator-activated receptor-*γ* agonists suppress the production of IL-12 family cytokines by activated glia. *Journal of Immunology*.

[35] Kielian T, McMahon M, Bearden ED, Baldwin AC, Drew PD, Esen N (2004). *S. aureus*-dependent microglial activation is selectively attenuated by the cyclopentenone prostaglandin 15-deoxy-Δ12,14-prostaglandin J2 (15d-PGJ2). *Journal of Neurochemistry*.

[36] Combs CK, Johnson DE, Karlo JC, Cannady SB, Landreth GE (2000). Inflammatory mechanisms in Alzheimer's disease: inhibition of *β*-amyloid-stimulated proinflammatory responses and neurotoxicity by PPAR*γ* agonists. *Journal of Neuroscience*.

[37] Kim EJ, Kwon KJ, Park J-Y, Lee SH, Moon C-H, Baik EJ (2002). Effects of peroxisome proliferator-activated receptor agonists on LPS-induced neuronal death in mixed cortical neurons: associated with iNOS and COX-2. *Brain Research*.

[38] Zhao X, Ou Z, Grotta JC, Waxham N, Aronowski J (2006). Peroxisome-proliferator-activated receptor-gamma (PPAR*γ*) activation protects neurons from NMDA excitotoxicity. *Brain Research*.

[39] Brodbeck J, Balestra ME, Saunders AM, Roses AD, Mahley RW, Huang Y (2008). Rosiglitazone increases dendritic spine density and rescues spine loss caused by apolipoprotein E4 in primary cortical neurons. *Proceedings of the National Academy of Sciences of the United States of America*.

[40] Smith SA, Monteith GR, Holman NA, Robinson JA, May FJ, Roberts-Thomson SJ (2003). Effects of peroxisome proliferator-activated receptor *γ* ligands ciglitazone and 15-deoxy-Δ^12,14^-prostaglandin J_2_ on rat cultured cerebellar granule neuronal viability. *Journal of Neuroscience Research*.

[41] Inestrosa NC, Godoy JA, Quintanilla RA, Koenig CS, Bronfman M (2005). Peroxisome proliferator-activated receptor *γ* is expressed in hippocampal neurons and its activation prevents *β*-amyloid neurodegeneration: role of Wnt signaling. *Experimental Cell Research*.

[42] Fuenzalida K, Quintanilla R, Ramos P (2007). Peroxisome proliferator-activated receptor *γ* up-regulates the Bcl-2 anti-apoptotic protein in neurons and induces mitochondrial stabilization and protection against oxidative stress and apoptosis. *Journal of Biological Chemistry*.

[43] Wada K, Nakajima A, Katayama K (2006). Peroxisome proliferator-activated receptor *γ*-mediated regulation of neural stem cell proliferation and differentiation. *Journal of Biological Chemistry*.

[44] Paintlia AS, Paintlia MK, Singh I, Singh AK (2006). IL-4-induced peroxisome proliferator-activated receptor *γ* activation inhibits NF-*κ*B trans activation in central nervous system (CNS) glial cells and protects oligodendrocyte progenitors under neuroinflammatory disease conditions: implication for CNS-demyelinating diseases. *Journal of Immunology*.

[45] Roth AD, Leisewitz AV, Jung JE (2003). PPAR *γ* activators induce growth arrest and process extension in B12 oligodendrocyte-like cells and terminal differentiation of cultured oligodendrocytes. *Journal of Neuroscience Research*.

[46] Clark RB, Bishop-Bailey D, Estrada-Hernandez T, Hla T, Puddington L, Padula SJ (2000). The nuclear receptor PPAR*γ* and immunoregulation: PPAR*γ* mediates inhibition of helper T cell responses. *Journal of Immunology*.

[47] Harris SG, Phipps RP (2001). The nuclear receptor PPAR gamma is expressed by mouse T lymphocytes and PPAR gamma agonists induce apoptosis. *European Journal of Immunology*.

[48] Jo S-H, Yang C, Miao Q (2006). Peroxisome proliferator-activated receptor *γ* promotes lymphocyte survival through its actions on cellular metabolic activities. *Journal of Immunology*.

[49] Hontecillas R, Bassaganya-Riera J (2007). Peroxisome proliferator-activated receptor *γ* is required for regulatory CD4^+^ T cell-mediated protection against colitis. *Journal of Immunology*.

[50] Wohlfert EA, Nichols FC, Nevius E, Clark RB (2007). Peroxisome proliferator-activated receptor *γ* (PPAR*γ*) and immunoregulation: enhancement of regulatory T cells through PPAR*γ*- dependent and -independent mechanisms. *Journal of Immunology*.

[51] Padilla J, Kaur K, Cao HJ, Smith TJ, Phipps RP (2000). Peroxisome proliferator activator receptor-*γ* agonists and 15-deoxy-Δ^12,14^-PGJ_2_ induce apoptosis in normal and malignant B-lineage cells. *Journal of Immunology*.

[52] Padilla J, Leung E, Phipps RP (2002). Human B lymphocytes and B lymphomas express PPAR-*γ* and are killed by PPAR-*γ* agonists. *Clinical Immunology*.

[53] Castrillo A, Tontonoz P (2004). Nuclear receptors in macrophage biology: at the crossroads of lipid metabolism and inflammation. *Annual Review of Cell and Developmental Biology*.

[54] Manuel SL, Rahman S, Wigdahl B, Khan ZK, Jain P (2007). Dendritic cells in autoimmune diseases and neuroinflammatory disorders. *Frontiers in Bioscience*.

[55] Wu GF, Laufer TM (2007). The role of dendritic cells in multiple sclerosis. *Current Neurology and Neuroscience Reports*.

[56] Szatmari I, Rajnavolgyi E, Nagy L (2006). PPAR*γ*, a lipid-activated transcription factor as a regulator of dendritic cell function. *Annals of the New York Academy of Sciences*.

[57] Nencioni A, Lauber K, Grünebach F (2002). Cyclopentenone prostaglandins induce caspase activation and apoptosis in dendritic cells by a PPAR-*γ*-independent mechanism: regulation by inflammatory and T cell-derived stimuli. *Experimental Hematology*.

[58] Angeli V, Hammad H, Staels B, Capron M, Lambrecht BN, Trottein F (2003). Peroxisome proliferator-activated receptor *γ* inhibits the migration of dendritic cells: consequences for the immune response. *Journal of Immunology*.

[59] Appel S, Mirakaj V, Bringmann A, Weck MM, Grünebach F, Brossart P (2005). PPAR-*γ* agonists inhibit toll-like receptor-mediated activation of dendritic cells via the MAP kinase and NF-*κ*B pathways. *Blood*.

[60] Klotz L, Dani I, Edenhofer F (2007). Peroxisome proliferator-activated receptor *γ* control of dendritic cell function contributes to development of CD4^+^ T cell anergy. *Journal of Immunology*.

[61] Godessart N, Kunkel SL (2001). Chemokines in autoimmune disease. *Current Opinion in Immunology*.

[62] Trebst C, Ransohoff RM (2001). Investigating chemokines and chemokine receptors in patients with multiple sclerosis: opportunities and challenges. *Archives of Neurology*.

[63] Kintscher U, Goetze S, Wakino S (2000). Peroxisome proliferator-activated receptor and retinoid X receptor ligands inhibit monocyte chemotactic protein-1-directed migration of monocytes. *European Journal of Pharmacology*.

[64] Zhang X, Wang JM, Gong WH, Mukaida N, Young HA (2001). Differential regulation of chemokine gene expression by 15-deoxy-Δ^12,14^ prostaglandin J_2_
^1,2^. *Journal of Immunology*.

[65] Marx N, Mach F, Sauty A (2000). Peroxisome proliferator-activated receptor-*γ* activators inhibit IFN-*γ*- induced expression of the T cell-active CXC chemokines IP-10, Mig, and I-TAC in human endothelial cells. *Journal of Immunology*.

[66] Chen N-G, Sarabia SF, Malloy PJ, Zhao X-Y, Feldman D, Reaven GM (1999). PPAR*γ* agonists enhance human vascular endothelial adhesiveness by increasing ICAM-1 expression. *Biochemical and Biophysical Research Communications*.

[67] Jackson SM, Parhami F, Xi X-P (1999). Peroxisome proliferator-activated receptor activators target human endothelial cells to inhibit leukocyte-endothelial cell interaction. *Arteriosclerosis, Thrombosis, and Vascular Biology*.

[68] Chen N-G, Han X (2001). Dual function of troglitazone in ICAM-1 gene expression in human vascular endothelium. *Biochemical and Biophysical Research Communications*.

[69] Maeshiba Y, Kiyota Y, Yamashita K, Yoshimura Y, Motohashi M, Tanayama S (1997). Disposition of the new antidiabetic agent pioglitazone in rats, dogs, and monkeys. *Arzneimittel-Forschung*.

[70] Risner ME, Saunders AM, Altman JFB (2006). Efficacy of rosiglitazone in a genetically defined population with mild-to-moderate Alzheimer's disease. *Pharmacogenomics Journal*.

[71] Niino M, Iwabuchi K, Kikuchi S (2001). Amelioration of experimental autoimmune encephalomyelitis in C57BL/6 mice by an agonist of peroxisome proliferator-activated receptor-*γ*. *Journal of Neuroimmunology*.

[72] Feinstein DL, Galea E, Gavrilyuk V (2002). Peroxisome proliferator-activated receptor-*γ* agonists prevent experimental autoimmune encephalomyelitis. *Annals of Neurology*.

[73] Natarajan C, Bright JJ (2002). Peroxisome proliferator-activated receptor-gamma agonist inhibit experimental allergic encephalomyelitis by blocking IL-12 production, IL-12 signaling and Th1 differentiation. *Genes & Immunity*.

[74] Natarajan C, Muthian G, Barak Y, Evans RM, Bright JJ (2003). Peroxisome proliferator-activated receptor-*γ*-deficient heterozygous mice develop an exacerbated neural antigen-induced Th1 response and experimental allergic encephalomyelitis. *Journal of Immunology*.

[75] Raikwar HP, Muthian G, Rajasingh J, Johnson C, Bright JJ (2005). PPAR*γ* antagonists exacerbate neural antigen-specific Th1 response and experimental allergic 
encephalomyelitis. *Journal of Neuroimmunology*.

[76] Schmidt S, Moric E, Schmidt M, Sastre M, Feinstein DL, Heneka MT (2004). Anti-inflammatory and antiproliferative actions of PPAR-*γ* agonists on T lymphocytes derived from MS patients. *Journal of Leukocyte Biology*.

[77] Klotz L, Schmidt M, Giese T (2005). Proinflammatory stimulation and pioglitazone treatment regulate peroxisome proliferator-activated receptor *γ* levels in peripheral blood mononuclear cells from healthy controls and multiple sclerosis patients. *Journal of Immunology*.

[78] Pershadsingh HA, Heneka MT, Saini R, Amin NM, Broeske DJ, Feinstein DL (2004). Effect of pioglitazone treatment in a patient with secondary multiple sclerosis. *Journal of Neuroinflammation*.

